# Evaluation of essential nursing care for children in the face of floods: a scoping review study 

**Published:** 2022-01

**Authors:** Fatameh Hoseinzadeh Siboni, Kasra Mohebbi, Zahra Taheri Ezbarami

**Affiliations:** ^*a*^ Department of Nursing, School of Nursing and Midwifery, Guilan University of Medical Sciences, Rasht, Iran.

## Abstract

**Background:**

Flood constitutes almost half of the natural disasters occurring in the last ten years. Children are more vulnerable because of their unique physiological, psychological, and growth characteristics.

**Methods:**

The methodology of this scoping review is in accordance with the proposed Arksey and O'Malley method. The primary question of the study was nursing care of children in the face of flood. Using 12 keywords, related articles were extracted from the search engines. Ultimately 18 articles were selected for the final inspection.

**Results:**

Findings were classified into six questions: 1. Which psychological problems arise for children and their parents during and after floods? 2. What is the effect of flood on children’s education? 3. Which factors and actions can reduce the flood vulnerability? 4. What are the health problems for children and their families during and after the flood? 5. Which factors affect the consequences of floods? The findings showed that children are much more vulnerable than adults, the most important factors influencing parental education are economic status, gender, age, the parents’ occupation, and other factors such as schools’ buildings, and proximity to rivers. Children suffer from various mental and physical disorders, child abuse, lack of access to health services, malnutrition, and growth and education process during and after the flood.

**Conclusions:**

Older children are more affected, maybe due to their greater recall. The next factor is gender, which affects girls more than boys due to their greater self-confidence and presence in rehabilitation activities. Parents' jobs and the family sources of income are important, as the loss of flood-affected jobs such as agriculture threatens the family and children's well-being. Spirituality has been introduced as the protective factor of children from the destructive effects of flood. After a flood, children experience post-traumatic stress disorder, respiratory illness, educational problems, diarrhea, malaria, child abuse, and lack of health facilities. Some of these consequences are preventable. Solutions such as hospital development, drug security, pre-flood vaccination, the education obligation, and preventing child marriage can be effective. In addition, children can acquire the skills needed to manage the situation during a flood and to help parents through the training they receive before a flood occurs.

**Keywords:**

Nursing care, Complication, Disaster, Flood, Children

